# Supplementary material for: Comparison of antibiotic prescribing records in two UK primary care electronic health record systems: cohort study using CPRD GOLD and CPRD Aurum databases
Source: BMJ Open. 2020 Jun 22;10(6):e038767. doi: 10.1136/bmjopen-2020-038767 (PMC7311011; doi:10.1136/bmjopen-2020-038767)
Supplement: Author's manuscript [file bmjopen-2020-038767.draft_revisions.pdf]

BMJ Open

BMJ Open is committed to open peer review. As part of this commitment we make the peer review history of every article we publish publicly available.

When an article is published we post the peer reviewers' comments and the authors' responses online. We also post the versions of the paper that were used during peer review. These are the versions that the peer review comments apply to.

The versions of the paper that follow are the versions that were submitted during the peer review process. They are not the versions of record or the final published versions. They should not be cited or distributed as the published version of this manuscript.

BMJ Open is an open access journal and the full, final, typeset and author-corrected version of record of the manuscript is available on our site with no access controls, subscription charges or pay-per-view fees (http://bmjopen.bmj.com).

If you have any questions on BMJ Open's open peer review process please email info.bmjopen@bmj.com

BMJ Open

Antibiotic prescribing records in two UK primary care electronic health record systems. Comparison of the CPRD GOLD and CPRD Aurum databases

Journal:	*BMJ Open*
Manuscript ID	bmjopen-2020-038767
Article Type:	Original research
Date Submitted by the Author:	25-Mar-2020
Complete List of Authors:	Gulliford, Martin; King's College London, School of Population Health and Environmental Sciences<br>Sun, Xiaohui; King's College London, School of Population Health and Environmental Sciences<br>Anjuman, Thamina; Clinical Practice Research Datalink, Medicines and Healthcare Products Regulatory Agency, London, UK<br>Yelland, Eleanor; Clinical Practice Research Datalink, Medicines and Healthcare Products Regulatory Agency, London, UK<br>Murray-Thomas, Tarita; Clinical Practice Research Datalink, Medicines and Healthcare Products Regulatory Agency, London, UK
Keywords:	PRIMARY CARE, Health informatics < BIOTECHNOLOGY & BIOINFORMATICS, EPIDEMIOLOGY, Public health < INFECTIOUS DISEASES

SCHOLARONE™
Manuscripts

BMJ

I, the Submitting Author has the right to grant and does grant on behalf of all authors of the Work (as defined in the below author licence), an exclusive licence and/or a non-exclusive licence for contributions from authors who are: i) UK Crown employees; ii) where BMJ has agreed a CC-BY licence shall apply, and/or iii) in accordance with the terms applicable for US Federal Government officers or employees acting as part of their official duties; on a worldwide, perpetual, irrevocable, royalty-free basis to BMJ Publishing Group Ltd ("BMJ") its licensees and where the relevant Journal is co-owned by BMJ to the co-owners of the Journal, to publish the Work in this journal and any other BMJ products and to exploit all rights, as set out in our licence.

The Submitting Author accepts and understands that any supply made under these terms is made by BMJ to the Submitting Author unless you are acting as an employee on behalf of your employer or a postgraduate student of an affiliated institution which is paying any applicable article publishing charge ("APC") for Open Access articles. Where the Submitting Author wishes to make the Work available on an Open Access basis (and intends to pay the relevant APC), the terms of reuse of such Open Access shall be governed by a Creative Commons licence – details of these licences and which Creative Commons licence will apply to this Work are set out in our licence referred to above.

Other than as permitted in any relevant BMJ Author's Self Archiving Policies, I confirm this Work has not been accepted for publication elsewhere, is not being considered for publication elsewhere and does not duplicate material already published. I confirm all authors consent to publication of this Work and authorise the granting of this licence.

For peer review only

Antibiotic prescribing records in two UK primary care electronic health record systems. Comparison of the CPRD GOLD and CPRD Aurum databases

Martin C Gulliford,[a,b] Xiaohui Sun,[a] Thamina Anjuman[c], Eleanor Yelland[c], and Tarita Murray-Thomas[c]

[a]King's College London, School of Population Health and Environmental Sciences, London, UK;

[b]National Institute for Health Research Biomedical Research Centre at Guy's and St Thomas' National Health Service Foundation Trust, London UK

[C]Clinical Practice Research Datalink, Medicines and Healthcare Products Regulatory Agency, London, UK

Short title: Antibiotic prescribing and GP systems

Correspondence: Martin Gulliford

King's College London,

School of Population Health and Environmental Sciences,

Addison House, Guy's Campus, London, SE1 1UL, UK

Tel: +44 207 848 6641

Fax: +44 207 848 6620

Email: martin.gulliford@kcl.ac.uk

Word count:	Abstract	324 words
	Text	2,596 words
	Tables	2
	Figures	1

For peer review only

ABSTRACT

Objective: We evaluated whether recording of antibiotic prescribing across two primary care electronic health record (EHR) systems is similar. Data were analysed from the Clinical Practice Research Datalink (CPRD) databases: CPRD GOLD (Vision data) and CPRD Aurum (EMIS data).

Methods: Cohorts of patients were randomly sampled from both databases, stratifying by general practice, age-group and gender. All antibiotic prescriptions in 2017 were identified. Age- and sex-standardised antibiotic prescribing rates per 1,000 person years were calculated. Prescribing of individual antibiotic products and associated medical diagnosis recorded on the same date was also evaluated. English CPRD GOLD general practices were analysed as a subgroup, because all CPRD Aurum practices sampled were in England.

Results: There were 101,360 antibiotic prescriptions among 158,305 sampled patients at 883 CPRD Aurum practices, and 112,931 prescriptions among 160,394 sampled patients at 290 CPRD GOLD practices. The age- and sex-standardised antibiotic prescribing rate in 2017 was 512.6 (95% confidence interval 510.4 to 514.9) per 1,000 person years in CPRD Aurum and 584.3 (582.1 to 586.5) per 1,000 person years in CPRD GOLD [505.2 (501.6 to 508.9) per 1,000 person years if restricted to practices in England]. The 25 most frequently prescribed antibiotic products were similar in both databases. One or more medical codes were recorded on the same date as an antibiotic prescription for 72,989 (74%) prescriptions in CPRD Aurum, 84,756 (78%) in CPRD GOLD, and 28,471 (78%) for CPRD GOLD in England. Skin, respiratory and genito-urinary tract infections were recorded for 39,035 (40%) prescriptions in CPRD Aurum, 41,326 (38%) in CPRD GOLD, with 15,481 (42%) in English CPRD GOLD practices only.

Conclusion: Similar estimates for antibiotic prescribing and infection recording were found for both databases suggesting similar recording across EMIS and Vision systems. Future research on antimicrobial stewardship can be conducted in CPRD Aurum informed by previous results from CPRD GOLD. It may also be possible to combine CPRD GOLD and CPRD Aurum data in research on antibiotic prescribing.

Key words: primary care, electronic healthcare records, antibiotics, infections

For peer review only - http://bmjopen.bmj.com/site/about/guidelines.xhtml

Strengths and limitations of this study

- The study drew on two databases that include large nationwide samples of general practices with large registered populations
- We excluded general practices that migrated between software systems
- We employed consistent data definitions and analysis methods across the two databases
- We analysed data for a single 12-month period, it is possible that changes over time may differ between the two data sources
- The study only investigated antibiotic prescribing, and related clinical coding, further studies are needed to address other topics of clinical and public health concern

For peer review only

For peer review only - http://bmjopen.bmj.com/site/about/guidelines.xhtml

INTRODUCTION

Primary care electronic health records (EHRs) provide an important source of longitudinal population-based data for public health research and population health surveillance.(1) In the UK, several EHR databases collect de-identified data from general practices, which are responsible for providing a broad range of general medical services in the primary care setting. The CPRD GOLD database (1) and The Health Improvement Network (THIN) database (2) collect data from general practices that use the Vision practice system; data from EMIS practice systems have been historically collected by the QResearch database.(3) CPRD has now established a new database, CPRD Aurum (4) that also collects data from general practices using the EMIS practice system.

While the research community has nearly 30 years of experience of using Vision data from CPRD GOLD, with numerous studies reporting on data quality,(5, 6) less is known about the similarities and differences with data collected in the CPRD Aurum database. EHR systems may offer end-users a variety of options, and differing incentives, to code clinical data and record test results. It is therefore possible that analysis of Vision-derived data from CPRD GOLD and EMIS-derived data from CPRD Aurum may yield different findings when substantive research questions are addressed. However, the magnitude of any possible differences between these data sources are largely unexplored. This study aimed to compare results obtained from an analysis of CPRD GOLD and CPRD Aurum data for one exemplar, the recording of antibiotic prescribing.

Antibiotic prescribing in primary care has been the subject of increasing interest in recent years because of the growing awareness that unnecessary prescription of antibiotics in primary care is contributing to the problem of antimicrobial resistance.(7, 8) Research using CPRD GOLD (9, 10) and THIN, (11) showed that the antibiotic prescribing rate is between

For peer review only

500 and 600 antibiotic prescriptions per 1,000 patient years, with higher rates at the extremes of age. Nearly half of antibiotic prescriptions may be issued without a clear indication being recorded.(9, 11) The present analysis aimed to determine whether analysis of data from CPRD Aurum and CPRD GOLD provides similar estimates with respect to antibiotic prescription and recording.

METHODS

Data and participants

The CPRD GOLD database collects data from the four countries of the UK, with 30% of contributing practices located in England at the time of this study. The CPRD GOLD database has been well described (1) and the high quality of the data collected has been documented in many studies.(12) The October 2019 database release from which the study cohort was sampled for this analysis included data on 17.6 million patients, of whom 2.6 million were currently active The CPRD Aurum database is more recently established, and at the time of this study (June 2019 release) drew on data collected from general practices in England only, using the EMIS practice system.(4) The CPRD Aurum database from which patients were sampled included data on 883 general practices with 23.1 million patients, including 2.5 million currently active patients. The study required analysis of anonymised data, the protocol for the study received scientific and ethical approval from the CPRD Independent Scientific Advisory Committee (ISAC Protocol 19_110R).

A sample of 158,305 patients in CPRD Aurum was taken by randomly selecting ‘n’ patients from each stratum of general practice, gender and age-group. The value of n=9 was selected to provide an appropriate total sample size of just over 150,000. This sampling

For peer review only - http://bmjopen.bmj.com/site/about/guidelines.xhtml

approach ensured that each general practice was equally represented in the analysis and that age-specific rates would be estimated with equal precision. Age was calculated as the difference between year of birth and 2017. Age-groups were categorised as 0-4, 5-14, 15-24, 25-34, 35-44, 45-54, 55-64, 65-74, 75-84 and 85 years and over.

A comparison cohort of patients was extracted from the October 2019 release of CPRD GOLD using the online interface. In this release, there were 290 general practices contributing data to CPRD GOLD throughout 2017. A sample of 160,394 patients was taken by randomly selecting n=30 patients from each stratum of general practice, gender and age-group. Patients were required to have at least 12 months of follow-up in the database estimated as the difference between the latest of their registration start date and 01/01/2017, and the earliest of registration end, practice last collection date, CPRD derived death date and 31/12/2017. General practices that migrated from Vision to EMIS practice systems during 2017 were excluded.

Measures

We evaluated antibiotic prescribing for the year 2017 because this was the most recent complete year that we included in a larger study of antibiotic prescribing that we report elsewhere.(13) Antibiotics included all drugs from section 5.1 of the British National Formulary (BNF)(14) except anti-tuberculous, anti-lepromatous agents, and methenamine. The BNF includes the following categories of antibiotics: penicillins, cephalosporins (including carbapenems), tetracyclines, aminoglycosides, macrolides, clindamycin, sulfonamides (including combinations with trimethoprim), metronidazole and tinidazole, quinolones, drugs for urinary tract infection (nitrofurantoin), and other antibiotics.

For peer review only - http://bmjopen.bmj.com/site/about/guidelines.xhtml

For CPRD GOLD, we employed a list of 2,627 antibiotic drugs that were identified from searches of the CPRD GOLD product dictionary browser made by all authors. Searches were made on the drug substance name, product name, BNF chapter and BNF codes. To identify the corresponding products in CPRD Aurum, dm+d codes (the prescribing codes from the National Health Service dictionary of medicines and devices) associated with individual product codes in the CPRD GOLD dictionary browser were mapped to the corresponding dm+d codes in the CPRD Aurum product dictionary browser. A more complete search of the CPRD Aurum product dictionary browser was additionally undertaken on term, product name and drug substance. We also conducted searches using approximate string matching (`fuzzy matching`) to match the CPRD Aurum product name to the CPRD GOLD product name or drug substance name from the CPRD GOLD antibiotic code list. The 'agrep' command was used in the R program,(15) using the Levenshtein edit distance as a measure of approximateness. The resulting code list was edited manually, resulting in 896 CPRD Aurum product codes. CPRD Aurum product codes are up to 17 characters in length and the 'bit64' package in R was employed for data formatting and management.(16) Although more product codes were identified for the CPRD GOLD database, only 195 CPRD GOLD product codes for antibiotics, and 167 CPRD Aurum product codes were recorded during 2017.

Medical diagnoses were identified by searching the CPRD GOLD medical dictionary browser for Read terms and inspecting the associated Read chapter hierarchy. Separate codes lists were included for respiratory conditions, genito-urinary infections, skin infections and eye infections as reported previously.(9) The CPRD Aurum medical dictionary includes Read terms, Read codes and SNOMED codes. In order to utilise the same codes, lists developed for CPRD GOLD were subsequently mapped to CPRD Aurum by matching on Read codes. Evidence of infections were searched in the patient clinical and referral records in CPRD GOLD and in the observation tables in CPRD Aurum.

For peer review only

Analysis

Age-specific rates were estimated with 95% confidence intervals from the Poisson distribution. Age- and sex-standardised rates, and associated 95% confidence intervals, were calculated per 1,000 person-years using the 2013 European Standard Population as reference. Differences between databases were evaluated using Bland-Altman plots.(17)

Patient and Public Involvement

Patients or the public were not involved in the design, or conduct, or reporting, or dissemination plans of this component of our research.

RESULTS

Comparison of antibiotic prescribing rates

In the CPRD Aurum sample, there were 158,305 participants from 883 general practices with 101,360 antibiotic prescriptions during 2017. In the CPRD GOLD sample there were 160,394 patients from 290 general practices with 112,931 antibiotic prescriptions during 2017. The age- and sex-standardised antibiotic prescribing rate was 512.6 (95% confidence interval 510.4 to 514.9) per 1,000 PYs in CPRD Aurum and 584.3 (582.1 to 586.5) per 1,000 PYs in CPRD GOLD. The rate for CPRD GOLD practices in England was 505.2 (501.6 to 508.9) per 1,000 PYs, which is similar to the rate observed in CPRD Aurum.

Figure 1 presents age- and sex-specific antibiotic prescribing rates for 2017. Antibiotic prescribing was higher in children under five years, decreased until the teenage years, increased again especially in women, before increasing steadily into older ages. This pattern of association was observed in both CPRD Aurum and CPRD GOLD but estimates for

For peer review only

CPRD GOLD were generally slightly higher than for CPRD Aurum, but generally similar when restricted to CPRD GOLD general practices in England. The lower panel of Figure 1 provides a Bland-Altman plot that presents the difference (95% confidence interval) between CPRD GOLD and CPRD Aurum for all CPRD GOLD practices (blue) and CPRD GOLD practices in England (red). In men and women for all age groups, CPRD GOLD general practices generally had slightly higher antibiotic prescribing rates than CPRD Aurum, while CPRD GOLD general practices in England had similar antibiotic prescribing rates to CPRD Aurum.

Most frequently prescribed products

Table 1 presents data for the 25 most frequently prescribed antibiotic products. In CPRD Aurum, amoxicillin 500mg capsules, doxycycline 100mg capsules, flucloxacillin 500mg capsules, trimethoprim 200mg tablets and nitrofurantoin 100mg modified-release capsules represented the five most frequently prescribed products, accounting for 45% of all antibiotic prescriptions. In CPRD GOLD, there were more prescriptions for trimethoprim and fewer prescriptions for nitrofurantoin, consequently clarithromycin 500 mg tablets and not nitrofurantoin appeared as the fifth ranked product. The same pattern was observed for CPRD GOLD practices in England, although trimethoprim comprised a smaller proportion of all prescriptions than in CPRD GOLD as a whole. Twenty-three of the 25 most frequently prescribed drugs in CPRD Aurum were also in the top 25 ranked prescriptions in CPRD GOLD general practices.

Recording of medical terms associated with prescriptions

Table 2 summarises data for recording of medical diagnostic codes on the same date as antibiotic prescriptions. Medical codes were recorded on the same date for 72,989 (74%) antibiotic prescriptions in CPRD Aurum, 84,756 (78%) antibiotic prescriptions in CPRD

For peer review only - http://bmjopen.bmj.com/site/about/guidelines.xhtml

For peer review only

GOLD and 28,471 (78%) for CPRD GOLD in England. Infections of the skin, respiratory tract and genito-urinary tracts, accounted for 39,035 (40%) of CPRD Aurum prescriptions, 41,326 (38%) of CPRD GOLD prescriptions and 15,481 (42%) of CPRD GOLD prescriptions in England. All other medical codes accounted for 33,954 (34%) in CPRD Aurum, 43,430 (40%) in CPRD GOLD and 12,990 (36%) CPRD GOLD in England.

DISCUSSION

Main findings

This analysis shows that antibiotic prescribing estimates from EMIS-derived data in CPRD Aurum are similar to those obtained through analysis of Vision-derived data in CPRD GOLD. This similarity includes the rates of antibiotic prescriptions for sub-groups of age and gender, the drug name and strength of antibiotic products prescribed, and the recording of medical diagnoses on the same day as the antibiotic prescription. We noted that antibiotic prescribing rates are generally higher in CPRD GOLD than in CPRD Aurum, but this was not the case when the CPRD GOLD sample was restricted to general practices in England. This implies that antibiotic prescribing may be higher in Wales, Scotland and Northern Ireland where there are no charges to patients for prescriptions. As well as slight differences in overall rates, we noted that drug choice might vary between databases. Trimethoprim prescribing was higher in CPRD GOLD than in CPRD Aurum. Nitrofurantoin has been recommended by Public Health England (18) as the drug of first choice for urinary tract infections in adults because of increasing antimicrobial resistance to trimethoprim. CPRD GOLD general practices in England were more similar to CPRD Aurum with respect to prescribing of trimethoprim and nitrofurantoin. It is likely that differences in clinical practice between England and the devolved administrations (Scotland, Wales and Northern Ireland) may be greater than the differences between EMIS and Vision practices within England.

For peer review only

Previous primary care database studies have revealed that antibiotic prescriptions are often issued without specific reasons being coded into electronic health records. A high proportion of antibiotic prescriptions in the THIN (11) and CPRD GOLD (9) databases are associated with either no code being recorded or non-specific codes being recorded. Electronic health records systems may offer users discretion over the recording of data items. We found that EMIS data included similar proportions of antibiotic prescriptions being associated with no codes, non-specific codes and codes for infection episodes.

Strength and Limitations

One major strength of our study is that we used real world data from primary care to estimate rates of antibiotic prescribing. Using data from primary care is likely to provide a reliable picture of prescribing patterns given that about 80% of all antibiotic prescribing in the UK's National Health Service take place in primary care.(18) We estimated the difference between CPRD Aurum and CPRD GOLD, as well as the difference between CPRD Aurum and CPRD GOLD in England. There were generally only small differences between CPRD Aurum and CPRD GOLD in England. We acknowledge that we could have obtained greater precision with larger samples, but the present approach was pragmatic and provided sufficiently precise estimates for age-specific rates. The community of CPRD researchers collectively have wide experience of compiling code lists for research in the CPRD GOLD database. Less experience is available for the CPRD Aurum database. We noted that CPRD Aurum product codes may be up to 17 characters in length and use of special programming features, such as the 'bit64' package in R,(16) are required in order to maintain data integrity. We completed extensive searches for product codes in order to identify antibiotic products. We identified a greater number of potential products from the CPRD GOLD data dictionary, but a generally similar number of antibiotic product codes were actually recorded in the two datasets during 2017. Searches in the CPRD Aurum product dictionary should be based on term, drug substance and product names as the BNF classification is less widely

For peer review only - http://bmjopen.bmj.com/site/about/guidelines.xhtml

For peer review only

available in the CPRD Aurum product dictionary than in CPRD GOLD. It may also be possible to compare 'dm+d' codes from the dictionary of medicines and devices, which are now employed in both CPRD GOLD and CPRD Aurum product dictionaries. We mapped medical code sets between CPRD GOLD and CPRD Aurum by matching on Read codes to make a like-for-like comparison. The analysis shows that, for these conditions, use of the same Read codes gives similar results in CPRD Aurum and CPRD GOLD. There are some medical codes that are only employed in EMIS, which might be omitted through this process, and this merits further evaluation. Experience shows that in Read-coded data, the majority of events are associated with a small number of codes, consequently omission of infrequently used codes is seldom important; our main findings with respect to medical codes were consistent between databases. We also note that records of antibiotic prescribing do not indicate whether medicines were dispensed, whether they were taken, or whether they were taken by the patient they were prescribed to or by someone else. It is also possible that prescriptions recorded at out of hours visits, home visits or during attendance at residential care homes may be missing from the patient electronic record. Finally, the analysis undertaken was cross-sectional in nature and does not provide evidence about trends in antibiotic recording over time between CPRD GOLD and CPRD Aurum. We used data from July and October 2019 releases of CPRD Aurum and CPRD Gold respectively, it may be preferable to compare the same month's releases but data for 2017 should be complete by 2019.

Conclusion

This study finds that analysis of EMIS-derived data in CPRD Aurum gives generally similar estimates for antibiotic prescribing and infection recording to those reported for Vision-derived data in CPRD GOLD. CPRD GOLD includes general practices in Scotland, Wales and Northern Ireland which have slightly higher antibiotic prescribing than either EMIS or Vision general practices in England. Based on these results, we believe that future research

For peer review only - http://bmjopen.bmj.com/site/about/guidelines.xhtml

studies can be conducted in CPRD Aurum, informed by previous results from CPRD GOLD or THIN. It may be possible to combine CPRD GOLD and CPRD Aurum data in research on antibiotic prescribing. However, further work is needed in other clinical topics and to understand the quality and completeness of information recorded in areas such as dosing regimen and treatment duration which is important in estimating treatment exposure in pharmacoepidemiology and pharmacovigilance research.

Acknowledgement

Data sources

The study is based on data from the Clinical Practice Research Datalink obtained under license from the UK Medicines and Healthcare products Regulatory Agency. However, the interpretation and conclusions contained in this report are those of the authors alone.

Data sharing

Requests for access to data from the study should be addressed to martin.gulliford@kcl.ac.uk. All proposals requesting data access will need to specify planned uses with approval of the study team and CPRD before data release.

Funding

The study is funded by the National Institute for Health Research (NIHR) Health Services and Delivery Programme (16/116/46). MG was supported by the NIHR Biomedical Research Centre at Guy’s and St Thomas’ Hospitals. The views expressed are those of the authors and not necessarily those of the NHS, the NIHR, or the Department of Health. The funder of the study had no role in study design, data collection, data analysis, data interpretation, or writing of the report. The authors had full access to all the data in the study and all authors shared final responsibility for the decision to submit for publication.

For peer review only - http://bmjopen.bmj.com/site/about/guidelines.xhtml

Conflict of Interest

The authors have no conflicts of interest.

Author Contributions

All authors contributed to the study design and definition of code lists. MG completed the analysis and drafted the paper. All authors reviewed and contributed to the final draft. MG is guarantor.

For peer review only

REFERENCES

1. Herrett E, Gallagher AM, Bhaskaran K, Forbes H, Mathur R, van Staa T, et al. Data Resource Profile: Clinical Practice Research Datalink (CPRD). *Int J Epidemiol.* 2015;**44(**3):827-36.

2. Petersen I, Welch CA, Nazareth I, Walters K, Marston L, Morris RW, et al. Health indicator recording in UK primary care electronic health records: key implications for handling missing data. *Clin Epidemiol.* 2019;**11**:157-67.

3. Hippisley-Cox J, Coupland C, Brindle P. The performance of seven QPrediction risk scores in an independent external sample of patients from general practice: a validation study. *BMJ Open*. 2014;4: e005809

4. Wolf A, Dedman D, Campbell J, Booth H, Lunn D, Chapman J, et al. Data resource profile: Clinical Practice Research Datalink (CPRD) Aurum. *Int J Epidemiol*. 2019; **48(**6):1740-1740g.

5. Herrett E, Thomas SL, Schoonen WM, Smeeth L, Hall AJ. Validation and validity of diagnoses in the General Practice Research Database: a systematic review. *Br J Clin Pharmacol.* 2010;**69**(1):4-14.

For peer review only - http://bmjopen.bmj.com/site/about/guidelines.xhtml

6. Williams T, van Staa T, Puri S, Eaton S. Recent advances in the utility and use of the General Practice Research Database as an example of a UK Primary Care Data resource. *Therapeutic Adv Drug Safety*. 2012;**3**(2):89-99.

7. Chief Medical Officer. *Annual Report of the Cheif Medical Officer. Volume 2, 2011. Infections and the rise of antimicrobial resistance*. London: Department of Health, 2013.

8. The Review on Antimicrobial Resistance by Jim O'Neill. *Tackling drug-resistant infections globally: final report and recommendations.* London: Review on antimicrobial resistance, 2016.

9. Sun X, Gulliford MC. Reducing antibiotic prescribing in primary care in England from 2014 to 2017: Population-based cohort study. *BMJOpen*. 2019.

10. Gulliford MC, Dregan A, Moore MV, Ashworth M, van Staa T, McCann G, et al. Continued high rates of antibiotic prescribing to adults with respiratory tract infection: survey of 568 UK general practices. *BMJ Open*. 2014;**4**(10):5.

11. Dolk FCK, Pouwels KB, Smith DRM, Robotham JV, Smieszek T. Antibiotics in primary care in England: which antibiotics are prescribed and for which conditions? *J Antimicrobial Chemother*. 2018;**73**(suppl_2):ii2-ii10.

12. Herrett EL, Thomas SL, Smeeth L. Validity of diagnoses in the general practice research database. *Br J Gen Pract*. 2011**;61**(588):438-9.

13. Gulliford MC, Sun X, Charlton J, Winter JR, Bunce C, Boiko O, Fox R, Little P, Moore M, Hay AD, Ashworth M. Serious bacterial infections and antibiotic prescribing in primary care. Cohort study using electronic health records in the UK. *BMJ Open.* 2020; **10** (2) e036975; DOI: 10.1136/bmjopen-2020-036975.

14. Royal Pharmaceutical Society. Medicines Complete: *British National Formulary 2017* [Available from: https://www.medicinescomplete.com/mc/bnflegacy/current/]

For peer review only

For peer review only - http://bmjopen.bmj.com/site/about/guidelines.xhtml

15. R Core Development Team. *R: A language and environment for statistical computing.* Vienna, Austria: R Foundation for Statistical Computing; 2010.

16. Oehlschlägel J. *Package 'bit64'. A S3 Class for Vectors of 64bit Integers,* 2017 [Available from: https://cran.r-project.org/web/packages/bit64/bit64.pdf.

17. Bland JM, Altman DG. Statistical methods for measuring agreement between two methods of clinical measurement. *Lancet.* 1986;**1**:307-10.

18. Public Health England. *English Surveillance Programme for Antimicrobial Utilisation and Resistance (ESPAUR) Report 2017*. London: Public Health England, 2017.

For peer review only

For peer review only - http://bmjopen.bmj.com/site/about/guidelines.xhtml

Figure 1: Antibiotic prescribing rates in CPRD Aurum and CPRD GOLD by age-group and sex.

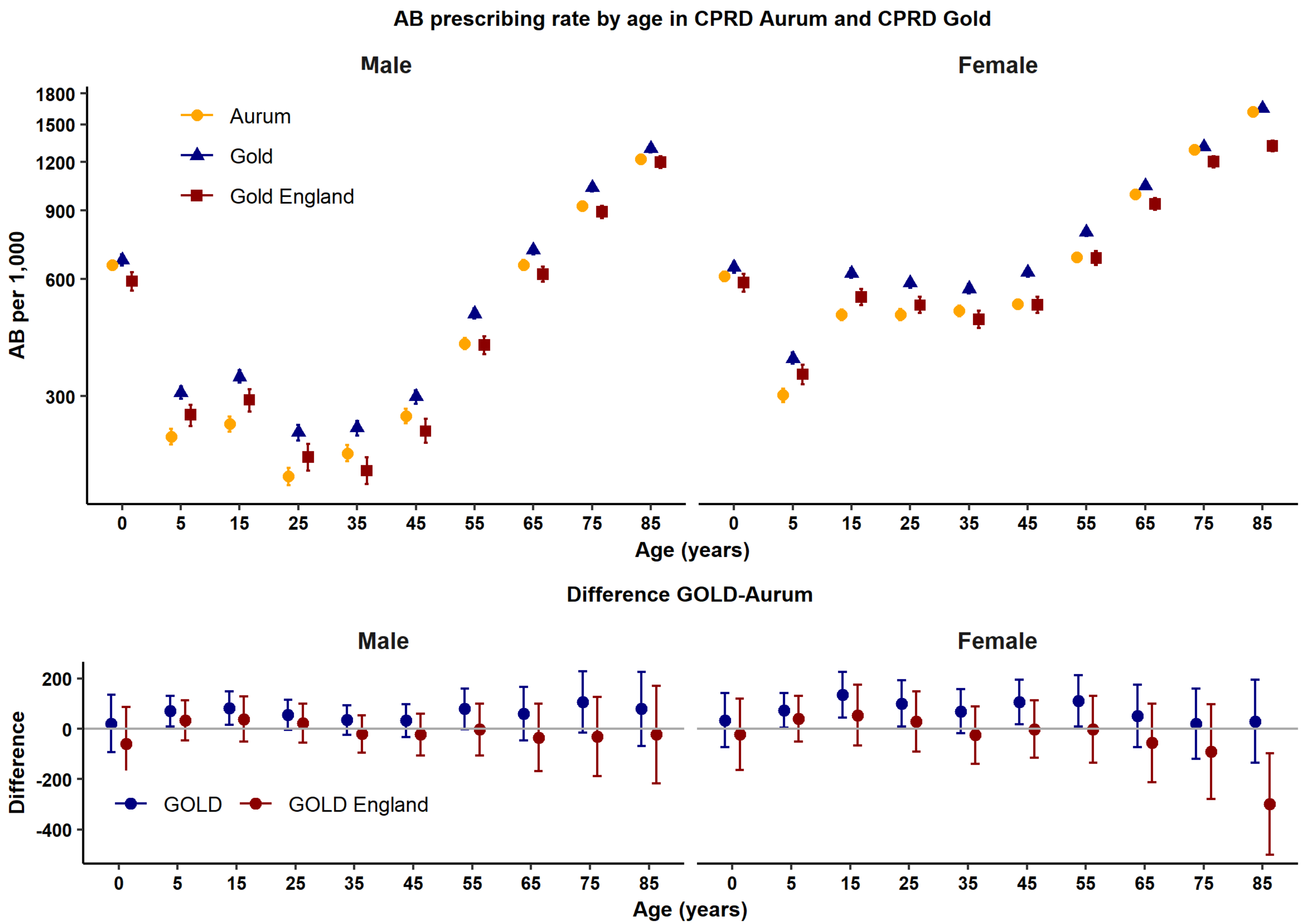

Table 1: Comparison of 25 most commonly prescribed antibiotic products in CPRD Aurum and CPRD GOLD.

Product Name	CPRD Aurum			CPRD GOLD			CPRD GOLD England		
	Freq.	Percent	Rank	Freq.	Percent	Rank	Freq.	Percent	Rank
Amoxicillin 500mg capsules	16,684	16.5	1	19,985	17.7	1	6,699	17.8	1
Doxycycline 100mg capsules	8,993	8.9	2	10,500	9.3	2	2,559	6.8	3
Flucloxacillin 500mg capsules	8,925	8.8	3	9,575	8.5	3	3,154	8.4	2
Trimethoprim 200mg tablets	5,931	5.9	4	9,064	8.0	4	2,277	6.1	4
Nitrofurantoin 100mg modified-release capsules	4,948	4.9	5	3,789	3.4	7	1,653	4.4	6
Clarithromycin 500mg tablets	4,656	4.6	6	4,889	4.3	5	1,842	4.9	5
Phenoxymethylpenicillin 250mg tablets	3,855	3.8	7	4,510	4.0	6	1,254	3.3	7
Amoxicillin 250mg/5ml oral suspension sugar free	3,030	3.0	8	3,645	3.2	8	1,065	2.8	9
Co-amoxiclav 500mg/125mg tablets	2,765	2.7	9	2,971	2.6	9	1,161	3.1	8
Lymecycline 408mg capsules	2,379	2.4	10	2,916	2.6	10	1,010	2.7	10
Nitrofurantoin 50mg capsules	2,295	2.3	11	2,821	2.5	11	848	2.3	12
Trimethoprim 100mg tablets	2,232	2.2	12	2,519	2.2	12	912	2.4	11
Nitrofurantoin 50mg tablets	2,169	2.1	13	1,539	1.4	18	610	1.6	17
Amoxicillin 250mg/5ml oral suspension	1,909	1.9	14	1,525	1.4	19	707	1.9	15
Amoxicillin 125mg/5ml oral suspension sugar free	1,802	1.8	15	990	0.9	25	369	1.0	25
Azithromycin 250mg tablets	1,602	1.6	16	1,306	1.2	22	373	1.0	24
Erythromycin 250mg gastro-resistant tablets	1,418	1.4	17	1,949	1.7	13	727	1.9	14
Metronidazole 400mg tablets	1,384	1.4	18	1,595	1.4	17	560	1.5	19
Oxytetracycline 250mg tablets	1,292	1.3	19	1,416	1.3	20	430	1.1	22
Cefalexin 250mg capsules	1,241	1.2	20	1,661	1.5	16	522	1.4	20
Ciprofloxacin 500mg tablets	1,236	1.2	21	1,724	1.5	15	662	1.8	16
Amoxil 500mg capsules (GlaxoSmithKline UK Ltd)	1,193	1.2	22	587	0.5	33	283	0.8	29
Flucloxacillin 250mg capsules	1,147	1.1	23	1,763	1.6	14	746	2.0	13
Phenoxymethylpenicillin 125mg/5ml oral solution	1,081	1.1	24	712	0.6	27	354	0.9	26
Amoxicillin 250mg capsules	1,073	1.1	25	1,397	1.2	21	575	1.5	18

For peer review only

Table 2: Medical coding of antibiotic prescriptions. Figures are frequencies (percent of unique prescription dates).

	CPRD Aurum	CPRD GOLD	CPRD GOLD England
Number of prescription items	101,360	112,931	37,551
Number of prescriptions with unique date	98,727	108,397	36,617
Medical code recorded on same date	72,989 (74.0)	84,756 (78.2)	28,471 (77.8)
No medical code recorded on same date	25,738 (26.0)	23,641 (21.8)	8,146 (22.2)
Respiratory infection	21,350 (21.6)	26,005 (24.0)	9,549 (26.1)
Genito-urinary infection	11,126 (11.3)	8,762 (8.1)	3,315 (9.1)
Skin infection	6,559 (6.6)	6,559 (6.1)	2,617 (7.1)
Other codes	33,954 (34.4)	43,430 (40.1)	12,990 (35.5)

For peer review only

For peer review only - http://bmjopen.bmj.com/site/about/guidelines.xhtml

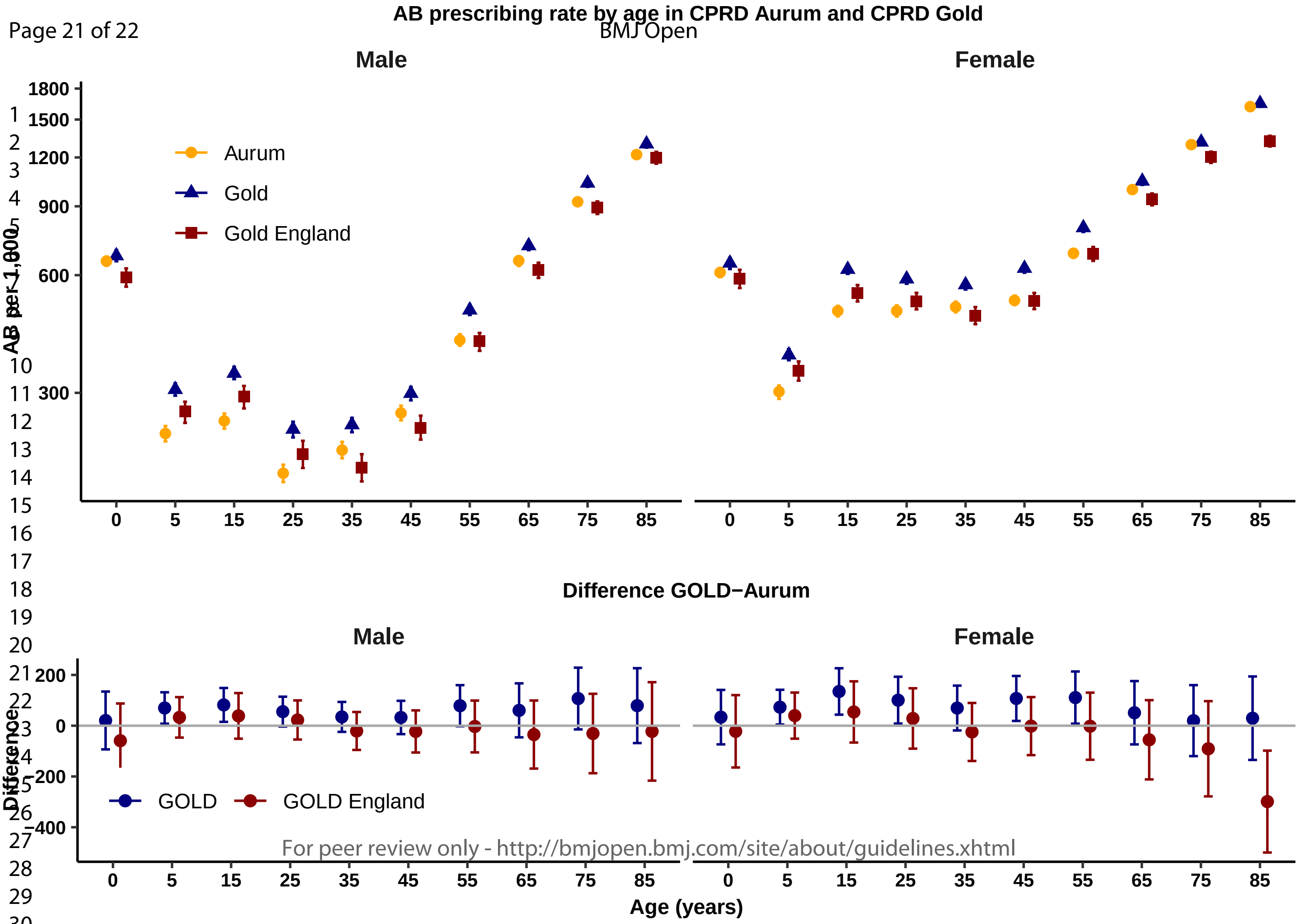

For peer review only - http://bmjopen.bmj.com/site/about/guidelines.xhtml

STROBE Statement—Checklist of items that should be included in reports of ***cohort studies***

	Item No	Recommendation
**Title and abstract**	1	(*a*) Indicate the study's design with a commonly used term in the title or the abstract **Abstract line 5**
		(*b*) Provide in the abstract an informative and balanced summary of what was done and what was found **Abstract**
**Introduction**		
Background/rationale	2	Explain the scientific background and rationale for the investigation being reported **P4**
Objectives	3	State specific objectives, including any prespecified hypotheses **P5**
**Methods**		
Study design	4	Present key elements of study design early in the paper **P5-6**
Setting	5	Describe the setting, locations, and relevant dates, including periods of recruitment, exposure, follow-up, and data collection **P5**
Participants	6	(*a*) Give the eligibility criteria, and the sources and methods of selection of participants. Describe methods of follow-up **P5-6**
		(*b*) For matched studies, give matching criteria and number of exposed and unexposed **N/A**
Variables	7	Clearly define all outcomes, exposures, predictors, potential confounders, and effect modifiers. Give diagnostic criteria, if applicable **P6-7**
Data sources/ measurement	8*	For each variable of interest, give sources of data and details of methods of assessment (measurement). Describe comparability of assessment methods if there is more than one group **P6-7**
Bias	9	Describe any efforts to address potential sources of bias **P8**
Study size	10	Explain how the study size was arrived at **P5-6**
Quantitative variables	11	Explain how quantitative variables were handled in the analyses. If applicable, describe which groupings were chosen and why **P6**
Statistical methods	12	(*a*) Describe all statistical methods, including those used to control for confounding **P8**
		(*b*) Describe any methods used to examine subgroups and interactions **N/A**
		(*c*) Explain how missing data were addressed **N/A**
		(*d*) If applicable, explain how loss to follow-up was addressed **N/A**
		(*e*) Describe any sensitivity analyses **N/A**
**Results**		
Participants	13*	(a) Report numbers of individuals at each stage of study—eg numbers potentially eligible, examined for eligibility, confirmed eligible, included in the study, completing follow-up, and analysed **P8**
		(b) Give reasons for non-participation at each stage **N/A**
		(c) Consider use of a flow diagram **N/A**
Descriptive data	14*	(a) Give characteristics of study participants (eg demographic, clinical, social) and information on exposures and potential confounders **P8**
		(b) Indicate number of participants with missing data for each variable of interest **Table 2**
		(c) Summarise follow-up time (eg, average and total amount) **P8**
Outcome data	15*	Report numbers of outcome events or summary measures over time **N/A**
Main results	16	(*a*) Give unadjusted estimates and, if applicable, confounder-adjusted estimates and their precision (eg, 95% confidence interval). Make clear which confounders were

For peer review only - http://bmjopen.bmj.com/site/about/guidelines.xhtml

		adjusted for and why they were included **N/A**
		(*b*) Report category boundaries when continuous variables were categorized **P7**
		(*c*) If relevant, consider translating estimates of relative risk into absolute risk for a meaningful time period **N/A**
Other analyses	17	Report other analyses done—eg analyses of subgroups and interactions, and sensitivity analyses **N/A**
**Discussion**		
Key results	18	Summarise key results with reference to study objectives **P10**
Limitations	19	Discuss limitations of the study, taking into account sources of potential bias or imprecision. Discuss both direction and magnitude of any potential bias **P11**
Interpretation	20	Give a cautious overall interpretation of results considering objectives, limitations, multiplicity of analyses, results from similar studies, and other relevant evidence **P12-13**
Generalisability	21	Discuss the generalisability (external validity) of the study results **P13**
**Other information**		
Funding	22	Give the source of funding and the role of the funders for the present study and, if applicable, for the original study on which the present article is based **P13**

*Give information separately for exposed and unexposed groups.

Note: An Explanation and Elaboration article discusses each checklist item and gives methodological background and published examples of transparent reporting. The STROBE checklist is best used in conjunction with this article (freely available on the Web sites of PLoS Medicine at http://www.plosmedicine.org/, Annals of Internal Medicine at http://www.annals.org/, and Epidemiology at http://www.epidem.com/). Information on the STROBE Initiative is available at http://www.strobe-statement.org.

For peer review only

For peer review only - http://bmjopen.bmj.com/site/about/guidelines.xhtml

BMJ Open

Comparison of antibiotic prescribing records in two UK primary care electronic health record systems: Cohort study using CPRD GOLD and CPRD Aurum databases

Journal:	*BMJ Open*
Manuscript ID	bmjopen-2020-038767.R1
Article Type:	Original research
Date Submitted by the Author:	07-May-2020
Complete List of Authors:	Gulliford, Martin; King's College London, School of Population Health and Environmental Sciences<br>Sun, Xiaohui; King's College London, School of Population Health and Environmental Sciences<br>Anjuman, Thamina; Clinical Practice Research Datalink, Medicines and Healthcare Products Regulatory Agency, London, UK<br>Yelland, Eleanor; Clinical Practice Research Datalink, Medicines and Healthcare Products Regulatory Agency, London, UK<br>Murray-Thomas, Tarita; Clinical Practice Research Datalink, Medicines and Healthcare Products Regulatory Agency, London, UK
**Primary Subject Heading**:	Epidemiology
Secondary Subject Heading:	General practice / Family practice
Keywords:	PRIMARY CARE, Health informatics < BIOTECHNOLOGY & BIOINFORMATICS, EPIDEMIOLOGY, Public health < INFECTIOUS DISEASES

SCHOLARONE™
Manuscripts

I, the Submitting Author has the right to grant and does grant on behalf of all authors of the Work (as defined in the below author licence), an exclusive licence and/or a non-exclusive licence for contributions from authors who are: i) UK Crown employees; ii) where BMJ has agreed a CC-BY licence shall apply, and/or iii) in accordance with the terms applicable for US Federal Government officers or employees acting as part of their official duties; on a worldwide, perpetual, irrevocable, royalty-free basis to BMJ Publishing Group Ltd ("BMJ") its licensees and where the relevant Journal is co-owned by BMJ to the co-owners of the Journal, to publish the Work in this journal and any other BMJ products and to exploit all rights, as set out in our licence.

The Submitting Author accepts and understands that any supply made under these terms is made by BMJ to the Submitting Author unless you are acting as an employee on behalf of your employer or a postgraduate student of an affiliated institution which is paying any applicable article publishing charge ("APC") for Open Access articles. Where the Submitting Author wishes to make the Work available on an Open Access basis (and intends to pay the relevant APC), the terms of reuse of such Open Access shall be governed by a Creative Commons licence – details of these licences and which Creative Commons licence will apply to this Work are set out in our licence referred to above.

Other than as permitted in any relevant BMJ Author's Self Archiving Policies, I confirm this Work has not been accepted for publication elsewhere, is not being considered for publication elsewhere and does not duplicate material already published. I confirm all authors consent to publication of this Work and authorise the granting of this licence.

For peer review only

Comparison of antibiotic prescribing records in two UK primary care electronic health record systems: Cohort study using CPRD GOLD and CPRD Aurum databases

Martin C Gulliford,[a,b] Xiaohui Sun,[a] Thamina Anjuman[c], Eleanor Yelland[c], and Tarita Murray-Thomas[c]

[a]King's College London, School of Population Health and Environmental Sciences, London, UK;

[b]National Institute for Health Research Biomedical Research Centre at Guy's and St Thomas' National Health Service Foundation Trust, London UK

[C]Clinical Practice Research Datalink, Medicines and Healthcare Products Regulatory Agency, London, UK

Short title: Antibiotic prescribing and GP systems

Correspondence: Martin Gulliford
King's College London,
School of Population Health and Environmental Sciences,
Addison House, Guy's Campus, London, SE1 1UL, UK
Tel: +44 207 848 6641
Fax: +44 207 848 6620

Email: martin.gulliford@kcl.ac.uk

Word count:

Abstract	293 words
Text	2,596 words
Tables	2
Figures	1

For peer review only

ABSTRACT

Objectives: We aimed to evaluate recording of antibiotic prescribing from two primary care electronic health record (EHR) systems.

Design: Cohort study.

Setting: UK general practices contributing to the Clinical Practice Research Datalink (CPRD) databases: CPRD GOLD (Vision data) and CPRD Aurum (EMIS data). English CPRD GOLD general practices were analysed as a subgroup, as all CPRD Aurum practices were located in England.

Participants: 158,305 patients were randomly sampled from CPRD Aurum and 160,394 from CPRD GOLD.

Outcome measures: Antibiotic prescriptions in 2017 were identified. Age- and sex-standardised antibiotic prescribing rates per 1,000 person years were calculated. Prescribing of individual antibiotic products and associated medical diagnoses were evaluated.

Results: There were 101,360 antibiotic prescriptions at 883 CPRD Aurum practices, and 112,931 prescriptions at 290 CPRD GOLD practices, including 112 general practices in England. The age- and sex-standardised antibiotic prescribing rate in 2017 was 512.6 (95% confidence interval 510.4 to 514.9) per 1,000 person years in CPRD Aurum and 584.3 (582.1 to 586.5) per 1,000 person years in CPRD GOLD [505.2 (501.6 to 508.9) per 1,000 person years if restricted to practices in England]. The 25 most frequently prescribed antibiotic products were similar in both databases. One or more medical codes were recorded on the same date as an antibiotic prescription for 72,989 (74%) prescriptions in CPRD Aurum, 84,756 (78%) in CPRD GOLD, and 28,471 (78%) for CPRD GOLD in England. Skin, respiratory and genito-urinary tract infections were recorded for 39,035 (40%) prescriptions in CPRD Aurum, 41,326 (38%) in CPRD GOLD, with 15,481 (42%) in English CPRD GOLD practices only.

Conclusion: Estimates for antibiotic prescribing and infection recording were broadly similar in both databases suggesting similar recording across EMIS and Vision systems. Future research on antimicrobial stewardship can also be conducted using primary care data in CPRD Aurum.

Key words: primary care, electronic healthcare records, antibiotics, infections, England, United Kingdom

For peer review only - http://bmjopen.bmj.com/site/about/guidelines.xhtml

Strengths and limitations of this study

- The study drew on two databases that include large nationwide samples of general practice registered populations.
- CPRD GOLD has UK-wide coverage and comprises data recorded using the Vision practice management system only. At the time of this study, CPRD Aurum general practices were located in England-only and included data recorded using the EMIS practice management system. This may contribute to differences between databases
- We excluded general practices that migrated from Vision to EMIS software system during the study period.
- We employed consistent data definitions and analysis methods across the two databases.
- We analysed data for a single 12-month period, it is possible that changes over time may differ between the two data sources
- The study only investigated antibiotic prescribing, and related clinical coding, further studies are needed to address other topics of clinical and public health concern

For peer review only - http://bmjopen.bmj.com/site/about/guidelines.xhtml

INTRODUCTION

Primary care electronic health records (EHRs) provide an important source of longitudinal population-based data for public health research and population health surveillance.(1) In the UK, several EHR databases collect de-identified data from general practices, which are responsible for providing a broad range of general medical services in the primary care setting. The CPRD GOLD database (1) and The Health Improvement Network (THIN) database (2) collect data from general practices that use the Vision practice system; data from EMIS practice systems have been historically collected by the QResearch database.(3) CPRD has now established a new database, CPRD Aurum (4) that also collects data from general practices using the EMIS practice system. In recent years, there has been a trend for general practices to switch from the Vision practice system to EMIS, with the latter increasing its market share. This has made evaluations of the quality of EMIS data increasingly important for research.

While the research community has nearly 30 years of experience of using Vision data from CPRD GOLD, with numerous studies reporting on data quality,(5, 6) less is known about the similarities and differences with data collected in the CPRD Aurum database. EHR systems may offer end-users a variety of options, and differing incentives, to code clinical data and record test results. It is therefore possible that analysis of Vision-derived data from CPRD GOLD and EMIS-derived data from CPRD Aurum may yield different findings when substantive research questions are addressed. However, the magnitude of any possible differences between these data sources are largely unexplored. This study aimed to compare results obtained from an analysis of CPRD GOLD and CPRD Aurum data for one exemplar, the recording of antibiotic prescribing.

For peer review only - http://bmjopen.bmj.com/site/about/guidelines.xhtml

Antibiotic prescribing in primary care has been the subject of increasing interest in recent years because of the growing awareness that unnecessary prescription of antibiotics in primary care is contributing to the problem of antimicrobial resistance.(7, 8) Research using CPRD GOLD (9, 10) and THIN, (11) showed that the antibiotic prescribing rate is between 500 and 600 antibiotic prescriptions per 1,000 patient years, with higher rates at the extremes of age. Nearly half of antibiotic prescriptions may be issued without a clear indication being recorded.(9, 11) The present analysis aimed to determine whether analysis of data from CPRD Aurum and CPRD GOLD provides similar estimates with respect to antibiotic prescription and recording.

METHODS

Data and participants

The CPRD GOLD database collects data from the four countries of the UK, with 30% of contributing practices located in England at the time of this study. The CPRD GOLD database has been well described (1) and the high quality of the data collected has been documented in many studies.(12) The October 2019 database release from which the study cohort was sampled for this analysis included data on 17.6 million patients, of whom 2.6 million were currently active. In this release, there were 320 currently contributing general practices including 30% in England, 3% in Northern Ireland, 37% in Scotland and 30% in Wales. The CPRD Aurum database is more recently established, and at the time of this study (June 2019 release) drew on data collected from general practices in England only, using the EMIS practice system.(4) The CPRD Aurum database included data on 883 general practices, from which patients were sampled, with 23.1 million patients, including 2.5 million currently active patients. The study required analysis of anonymised data, the

For peer review only - http://bmjopen.bmj.com/site/about/guidelines.xhtml

protocol for the study received scientific and ethical approval from the CPRD Independent Scientific Advisory Committee (ISAC Protocol 19_110R).

A sample of 158,305 patients in CPRD Aurum was taken by randomly selecting ‘n’ patients from each stratum of general practice, gender and age-group. The value of n=9 was selected to provide an appropriate total sample size of just over 150,000. This sampling approach ensured that each general practice was equally represented in the analysis and that age-specific rates would be estimated with equal precision. Age was calculated as the difference between year of birth and 2017. Age-groups were categorised as 0-4, 5-14, 15-24, 25-34, 35-44, 45-54, 55-64, 65-74, 75-84 and 85 years and over.

A comparison cohort of patients was extracted from the October 2019 release of CPRD GOLD using the online interface. In this release, there were 290 general practices contributing data to CPRD GOLD throughout 2017, including 112 in England. A sample of 160,394 patients was taken by randomly selecting n=30 patients from each stratum of general practice, gender and age-group. Patients were required to have at least 12 months of follow-up in the database estimated as the difference between the latest of their registration start date and 01/01/2017, and the earliest of registration end, practice last collection date, CPRD derived death date and 31/12/2017. General practices that migrated from Vision to EMIS practice systems during 2017 were excluded.

Measures

We evaluated antibiotic prescribing for the year 2017 because this was the most recent complete year that we included in a larger study of antibiotic prescribing that we report elsewhere.(13) We identified all antibiotic prescriptions issued in 2017, including all drugs

For peer review only

For peer review only - http://bmjopen.bmj.com/site/about/guidelines.xhtml

from section 5.1 of the British National Formulary (BNF)(14) except anti-tuberculous, anti-lepromatous agents, and methenamine. The BNF includes the following categories of antibiotics: penicillins, cephalosporins (including carbapenems), tetracyclines, aminoglycosides, macrolides, clindamycin, sulfonamides (including combinations with trimethoprim), metronidazole and tinidazole, quinolones, drugs for urinary tract infection (nitrofurantoin), and other antibiotics.

For CPRD GOLD, we employed a list of 2,627 antibiotic drugs that were identified from searches of the CPRD GOLD product dictionary browser made by all authors. Searches were made on the drug substance name, product name, BNF chapter and BNF codes. To identify the corresponding products in CPRD Aurum, dm+d codes (the prescribing codes from the National Health Service dictionary of medicines and devices) associated with individual product codes in the CPRD GOLD dictionary browser were mapped to the corresponding dm+d codes in the CPRD Aurum product dictionary browser. A more complete search of the CPRD Aurum product dictionary browser was additionally undertaken on term, product name and drug substance. We also conducted searches using approximate string matching (`fuzzy matching`) to match the CPRD Aurum product name to the CPRD GOLD product name or drug substance name from the CPRD GOLD antibiotic code list. The 'agrep' command was used in the R program,(15) using the Levenshtein edit distance as a measure of approximateness. The resulting code list was edited manually, resulting in 896 CPRD Aurum product codes. CPRD Aurum product codes are up to 17 characters in length and the 'bit64' package in R was employed for data formatting and management.(16) Although more product codes were identified for the CPRD GOLD database, only 195 CPRD GOLD product codes for antibiotics, and 167 CPRD Aurum product codes were recorded during 2017.

For peer review only

We analysed medical codes recorded on the same date as antibiotic prescriptions. Medical diagnoses were identified by searching the CPRD GOLD medical dictionary browser for Read terms and inspecting the associated Read chapter hierarchy. As previously reported, all medical codes were subsequently classified as respiratory infections, genito-urinary infections, skin infections, eye infections and “other codes” .(9) The CPRD Aurum medical dictionary includes Read terms, Read codes and SNOMED codes. In order to utilise the same codes, lists developed for CPRD GOLD were subsequently mapped to CPRD Aurum by matching on Read codes. Evidence of infections were searched in the patient clinical and referral records in CPRD GOLD and in the observation tables in CPRD Aurum. We evaluated whether or not any medical code was recorded on the same date as an antibiotic prescription. We then classified medical codes into ‘respiratory infections’, ‘skin infections’, ‘genito-urinary infections’, and ‘other codes’.

Analysis

Age-specific rates were estimated with 95% confidence intervals from the Poisson distribution. Age- and sex-standardised rates, and associated 95% confidence intervals, were calculated per 1,000 person-years using the 2013 European Standard Population as reference. Null hypothesis significance testing was not undertaken. Instead, we present estimates and 95% confidence intervals to enable the reader to judge whether differences between the databases may be of clinical or epidemiological importance. Potential differences between databases were evaluated using Bland-Altman plots and 95% confidence intervals (17) CPRD GOLD general practices in England were analysed as a sub-group.

For peer review only - http://bmjopen.bmj.com/site/about/guidelines.xhtml

Patient and Public Involvement

There was no patient or public involvement in the study including development of the research question, selection of outcome measures, study design, conduct or dissemination of findings.

RESULTS

Comparison of antibiotic prescribing rates

In the CPRD Aurum sample, there were 158,305 participants from 883 general practices with 101,360 antibiotic prescriptions during 2017. In the CPRD GOLD sample there were 160,394 patients from 290 general practices with 112,931 antibiotic prescriptions during 2017. This included 112 general practices in England and 178 in Scotland, Wales or Northern Ireland. The age- and sex-standardised antibiotic prescribing rate was 512.6 (95% confidence interval 510.4 to 514.9) per 1,000 PYs in CPRD Aurum and 584.3 (582.1 to 586.5) per 1,000 PYs in CPRD GOLD. The rate for CPRD GOLD practices in England was 505.2 (501.6 to 508.9) per 1,000 PYs, which is similar to the rate observed in CPRD Aurum.

Figure 1 presents age- and sex-specific antibiotic prescribing rates for 2017. Antibiotic prescribing was higher in children under five years, decreased until the teenage years, increased again especially in women, before increasing steadily into older ages. This pattern of association was observed in both CPRD Aurum and CPRD GOLD but estimates for CPRD GOLD were slightly higher than for CPRD Aurum, but broadly similar when restricted to CPRD GOLD general practices in England. The lower panel of Figure 1 provides a Bland-Altman plot that presents the difference (95% confidence interval) between all CPRD GOLD and CPRD Aurum practices (blue) and CPRD GOLD practices in England only (red). For men and women in all age groups, CPRD GOLD general practices generally had slightly

For peer review only - http://bmjopen.bmj.com/site/about/guidelines.xhtml

higher antibiotic prescribing rates than CPRD Aurum, while CPRD GOLD general practices in England had broadly similar antibiotic prescribing rates to CPRD Aurum. Confidence intervals were compatible with no difference in all except the oldest age-group (≥85 years of age) where data are more sparse.

Most frequently prescribed products

Table 1 presents data for the 25 most frequently prescribed antibiotic products. In CPRD Aurum, amoxicillin 500mg capsules, doxycycline 100mg capsules, flucloxacillin 500mg capsules, trimethoprim 200mg tablets and nitrofurantoin 100mg modified-release capsules represented the five most frequently prescribed products, accounting for 45% of all antibiotic prescriptions. In CPRD GOLD, there were more prescriptions for trimethoprim (8%) and fewer prescriptions for nitrofurantoin (3%), consequently clarithromycin 500 mg tablets and not nitrofurantoin appeared as the fifth ranked product. The same pattern was observed for CPRD GOLD practices in England, although trimethoprim comprised a smaller proportion of all prescriptions than in CPRD GOLD as a whole. Twenty-three of the 25 most frequently prescribed drugs in CPRD Aurum were also in the top 25 ranked prescriptions in CPRD GOLD general practices.

Recording of medical terms associated with prescriptions

Table 2 summarises data for recording of medical diagnostic codes on the same date as antibiotic prescriptions. Medical codes were recorded on the same date for 72,989 (74%) antibiotic prescriptions in CPRD Aurum, 84,756 (78%) antibiotic prescriptions in CPRD GOLD and 28,471 (78%) for CPRD GOLD in England. Infections of the skin, respiratory tract and genito-urinary tracts, accounted for 39,035 (40%) of CPRD Aurum prescriptions, 41,326 (38%) of CPRD GOLD prescriptions and 15,481 (42%) of CPRD GOLD prescriptions in

For peer review only - http://bmjopen.bmj.com/site/about/guidelines.xhtml

England. All other medical codes accounted for 33,954 (34%) in CPRD Aurum, 43,430 (40%) in CPRD GOLD and 12,990 (36%) CPRD GOLD in England.

DISCUSSION

Main findings

This analysis shows that antibiotic prescribing estimates from EMIS-derived data in CPRD Aurum are broadly similar to those obtained through analysis of Vision-derived data in CPRD GOLD. This similarity includes the rates of antibiotic prescriptions for sub-groups of age and gender, the drug name and strength of antibiotic products prescribed, and the recording of medical diagnoses on the same day as the antibiotic prescription. We noted that antibiotic were more frequently prescribed in CPRD GOLD than in CPRD Aurum, but this was not the case when the CPRD GOLD sample was restricted to general practices in England. This suggests that antibiotic prescribing may be higher in Wales, Northern Ireland and Scotland where prescription charges have been abolished since 2007, 2010 and 2011 respectively,(18, 19) although our study did not investigate the reasons for this difference. As well as slight differences in overall rates, we noted that drug choice might vary between databases. Trimethoprim prescribing was higher in CPRD GOLD than in CPRD Aurum. Nitrofurantoin has been recommended by Public Health England (20) as the drug of first choice for urinary tract infections in adults because of increasing antimicrobial resistance to trimethoprim but this guidance may not apply in the devolved administrations. CPRD GOLD general practices in England were more similar to CPRD Aurum with respect to prescribing of trimethoprim and nitrofurantoin. It is likely that differences in clinical practice between England and the devolved administrations (Scotland, Wales and Northern Ireland) may be greater than the differences between EMIS and Vision practices within England.

For peer review only - http://bmjopen.bmj.com/site/about/guidelines.xhtml

Previous primary care database studies have revealed that antibiotic prescriptions are often issued without specific reasons being coded into electronic health records. A high proportion of antibiotic prescriptions in the THIN (11) and CPRD GOLD (9) databases are associated with either no code being recorded or non-specific codes being recorded. Electronic health records systems may offer users discretion over the recording of data items. We found that EMIS data included similar proportions of antibiotic prescriptions being associated with no codes, non-specific codes and codes for infection episodes.

Strength and Limitations

One major strength of our study is that we used real world data from primary care to estimate rates of antibiotic prescribing. Using data from primary care is likely to provide a reliable picture of prescribing patterns given that about 80% of all antibiotic prescribing in the UK's National Health Service take place in primary care.(20) We estimated the difference between CPRD Aurum and CPRD GOLD, as well as the difference between CPRD Aurum and CPRD GOLD in England. A comparison between CPRD GOLD practices in England and CPRD Aurum practices was essential to benchmark recording in the CPRD Aurum database, which at the time of this study comprised of contributing practices from England only. We recommend that future comparative studies on antibiotic prescribing in the UK should separately evaluate prescribing in CPRD Aurum with CPRD GOLD practices in England, and CPRD GOLD practices in Wales, Scotland and Northern Ireland, given that factors such as socioeconomic differences, as well as abolished prescription charges, in the devolved nations may have an impact on prescribing in these countries. There were generally only small differences between CPRD Aurum and CPRD GOLD in England. We acknowledge that we could have obtained greater precision with larger samples, but the present approach was pragmatic and provided sufficiently precise estimates for age-specific rates. We did not employ null-hypothesis significance testing but elected to present confidence intervals so that readers could reflect on the substantive importance of any

For peer review only

estimated differences for their proposed studies. The community of CPRD researchers collectively have wide experience of compiling code lists for research in the CPRD GOLD database. Less experience is available for the CPRD Aurum database. We noted that CPRD Aurum product codes may be up to 17 characters in length and use of special programming features, such as the 'bit64' package in R,(16) are required in order to maintain data integrity. We completed extensive searches for product codes in order to identify antibiotic products. We identified a greater number of potential products from the CPRD GOLD data dictionary, but a generally similar number of antibiotic product codes were actually recorded in the two datasets during 2017. Searches in the CPRD Aurum product dictionary should be based on term, drug substance and product names as the BNF classification is less widely available in the CPRD Aurum product dictionary than in CPRD GOLD. It may also be possible to compare 'dm+d' codes from the dictionary of medicines and devices, which are now employed in both CPRD GOLD and CPRD Aurum product dictionaries. We mapped medical code sets between CPRD GOLD and CPRD Aurum by matching on Read codes to make a like-for-like comparison. The analysis shows that, for these conditions, use of the same Read codes gives similar results in CPRD Aurum and CPRD GOLD. There are some medical codes that are only employed in EMIS, which might be omitted through this process, and this merits further evaluation. Experience shows that in Read-coded data, the majority of events are associated with a small number of codes, consequently omission of infrequently used codes is seldom important; our main findings with respect to medical codes were consistent between databases. We also note that records of antibiotic prescribing do not indicate whether medicines were dispensed, whether they were taken, or whether they were taken by the patient they were prescribed to or by someone else. It is also possible that prescriptions recorded at out of hours visits, home visits or during attendance at residential care homes may be missing from the patient electronic record. Finally, the analysis undertaken was cross-sectional in nature and does not provide evidence about trends in antibiotic recording over time between CPRD GOLD and CPRD Aurum. We used data from July and October 2019 releases of CPRD Aurum and CPRD Gold respectively, it may be

For peer review only - http://bmjopen.bmj.com/site/about/guidelines.xhtml

preferable to compare the same month's releases but data for 2017 should be complete by 2019.

Conclusion

This study finds that analysis of EMIS-derived data in CPRD Aurum gives broadly similar estimates for antibiotic prescribing and infection recording to those reported for Vision-derived data in CPRD GOLD. CPRD GOLD includes general practices in Scotland, Wales and Northern Ireland which have slightly higher antibiotic prescribing than either EMIS or Vision general practices in England. Based on these results, we believe that future research studies can be conducted in CPRD Aurum, informed by previous results from CPRD GOLD or THIN. It may also be possible to combine data from CPRD GOLD English practices with CPRD Aurum data in research on antibiotic prescribing. As CPRD Aurum includes an increasing number of general practices this database will become increasingly important for public health research. However, further work is needed to better understand the quality and completeness of information recorded in areas such as dosing regimen and treatment duration which are important in estimating treatment exposure in pharmacoepidemiology and pharmacovigilance research.

For peer review only - http://bmjopen.bmj.com/site/about/guidelines.xhtml

Acknowledgement

Data sources

The study is based on data from the Clinical Practice Research Datalink obtained under license from the UK Medicines and Healthcare products Regulatory Agency. However, the interpretation and conclusions contained in this report are those of the authors alone.

Data sharing

Requests for access to data from the study should be addressed to martin.gulliford@kcl.ac.uk. All proposals requesting data access will need to specify planned uses with approval of the study team and CPRD before data release.

Funding

The study is funded by the National Institute for Health Research (NIHR) Health Services and Delivery Programme (16/116/46). MG was supported by the NIHR Biomedical Research Centre at Guy's and St Thomas' Hospitals. The views expressed are those of the authors and not necessarily those of the NHS, the NIHR, or the Department of Health. The funder of the study had no role in study design, data collection, data analysis, data interpretation, or writing of the report. The authors had full access to all the data in the study and all authors shared final responsibility for the decision to submit for publication.

Conflict of Interest

The authors have no conflicts of interest.

Author Contributions

MG designed the study. TA sampled CPRD Aurum data. XS developed code lists for CPRD GOLD. TA, EY and TMT checked and revised code lists for CPRD Aurum. MG completed the analysis and drafted the paper. All authors reviewed and contributed to the final draft. MG is guarantor.

REFERENCES

1. Herrett E, Gallagher AM, Bhaskaran K, Forbes H, Mathur R, van Staa T, et al. Data Resource Profile: Clinical Practice Research Datalink (CPRD). *Int J Epidemiol.* 2015;**44(**3):827-36.

2. Petersen I, Welch CA, Nazareth I, Walters K, Marston L, Morris RW, et al. Health indicator recording in UK primary care electronic health records: key implications for handling missing data. *Clin Epidemiol.* 2019;**11**:157-67.

3. Hippisley-Cox J, Coupland C, Brindle P. The performance of seven QPrediction risk scores in an independent external sample of patients from general practice: a validation study. *BMJ Open*. 2014;4: e005809

4. Wolf A, Dedman D, Campbell J, Booth H, Lunn D, Chapman J, et al. Data resource profile: Clinical Practice Research Datalink (CPRD) Aurum. *Int J Epidemiol*. 2019; **48(**6):1740-1740g.

5. Herrett E, Thomas SL, Schoonen WM, Smeeth L, Hall AJ. Validation and validity of diagnoses in the General Practice Research Database: a systematic review. *Br J Clin Pharmacol.* 2010;**69**(1):4-14.

6. Williams T, van Staa T, Puri S, Eaton S. Recent advances in the utility and use of the General Practice Research Database as an example of a UK Primary Care Data resource. *Therapeutic Adv Drug Safety*. 2012;**3**(2):89-99.

7. Chief Medical Officer. *Annual Report of the Cheif Medical Officer. Volume 2, 2011. Infections and the rise of antimicrobial resistance*. London: Department of Health, 2013.

8. The Review on Antimicrobial Resistance by Jim O'Neill. *Tackling drug-resistant infections globally: final report and recommendations.* London: Review on antimicrobial resistance, 2016.

For peer review only - http://bmjopen.bmj.com/site/about/guidelines.xhtml

9. Sun X, Gulliford MC. Reducing antibiotic prescribing in primary care in England from 2014 to 2017: Population-based cohort study. *BMJOpen*. 2019.

10. Gulliford MC, Dregan A, Moore MV, Ashworth M, van Staa T, McCann G, et al. Continued high rates of antibiotic prescribing to adults with respiratory tract infection: survey of 568 UK general practices. *BMJ Open*. 2014;**4**(10):5.

11. Dolk FCK, Pouwels KB, Smith DRM, Robotham JV, Smieszek T. Antibiotics in primary care in England: which antibiotics are prescribed and for which conditions? *J Antimicrobial Chemother*. 2018;**73**(suppl_2):ii2-ii10.

12. Herrett EL, Thomas SL, Smeeth L. Validity of diagnoses in the general practice research database. *Br J Gen Pract*. 2011**;61**(588):438-9.

13. Gulliford MC, Sun X, Charlton J, Winter JR, Bunce C, Boiko O, Fox R, Little P, Moore M, Hay AD, Ashworth M. Serious bacterial infections and antibiotic prescribing in primary care. Cohort study using electronic health records in the UK. *BMJ Open.* 2020; **10** (2) e036975; DOI: 10.1136/bmjopen-2020-036975.

14. Royal Pharmaceutical Society. Medicines Complete: *British National Formulary 2017* [Available from: https://www.medicinescomplete.com/mc/bnflegacy/current/]

15. R Core Development Team. *R: A language and environment for statistical computing.* Vienna, Austria: R Foundation for Statistical Computing; 2010.

16. Oehlschlägel J. *Package 'bit64'. A S3 Class for Vectors of 64bit Integers,* 2017 [Available from: https://cran.r-project.org/web/packages/bit64/bit64.pdf.

17. Bland JM, Altman DG. Statistical methods for measuring agreement between two methods of clinical measurement. *Lancet.* 1986;**1**:307-10.

For peer review only - http://bmjopen.bmj.com/site/about/guidelines.xhtml

433 18. Cohen D, Alam MF, Dunstan FD, Myles S, Hughes DA, Routledge PA. Abolition of prescription copayments in Wales: an observational study on dispensing rates. *Value Health*. 2010;**13**:675-80.

436 19. Williams AJ, Henley W, Frank J. Impact of abolishing prescription fees in Scotland on hospital admissions and prescribed medicines: an interrupted time series evaluation. *BMJ Open.* 2018;**8**:e021318.

439 20. Public Health England. *English Surveillance Programme for Antimicrobial Utilisation and Resistance (ESPAUR) Report 2017*. London: Public Health England, 2017.

441

For peer review only

Table 1: Comparison of 25 most commonly prescribed antibiotic products in CPRD Aurum and CPRD GOLD.

Product Name	CPRD Aurum			CPRD GOLD			CPRD GOLD England		
	Freq.	Percent	Rank	Freq.	Percent	Rank	Freq.	Percent	Rank
Amoxicillin 500mg capsules	16,684	16.5	1	19,985	17.7	1	6,699	17.8	1
Doxycycline 100mg capsules	8,993	8.9	2	10,500	9.3	2	2,559	6.8	3
Flucloxacillin 500mg capsules	8,925	8.8	3	9,575	8.5	3	3,154	8.4	2
Trimethoprim 200mg tablets	5,931	5.9	4	9,064	8.0	4	2,277	6.1	4
Nitrofurantoin 100mg modified-release capsules	4,948	4.9	5	3,789	3.4	7	1,653	4.4	6
Clarithromycin 500mg tablets	4,656	4.6	6	4,889	4.3	5	1,842	4.9	5
Phenoxymethylpenicillin 250mg tablets	3,855	3.8	7	4,510	4.0	6	1,254	3.3	7
Amoxicillin 250mg/5ml oral suspension sugar free	3,030	3.0	8	3,645	3.2	8	1,065	2.8	9
Co-amoxiclav 500mg/125mg tablets	2,765	2.7	9	2,971	2.6	9	1,161	3.1	8
Lymecycline 408mg capsules	2,379	2.4	10	2,916	2.6	10	1,010	2.7	10
Nitrofurantoin 50mg capsules	2,295	2.3	11	2,821	2.5	11	848	2.3	12
Trimethoprim 100mg tablets	2,232	2.2	12	2,519	2.2	12	912	2.4	11
Nitrofurantoin 50mg tablets	2,169	2.1	13	1,539	1.4	18	610	1.6	17
Amoxicillin 250mg/5ml oral suspension	1,909	1.9	14	1,525	1.4	19	707	1.9	15
Amoxicillin 125mg/5ml oral suspension sugar free	1,802	1.8	15	990	0.9	25	369	1.0	25
Azithromycin 250mg tablets	1,602	1.6	16	1,306	1.2	22	373	1.0	24
Erythromycin 250mg gastro-resistant tablets	1,418	1.4	17	1,949	1.7	13	727	1.9	14
Metronidazole 400mg tablets	1,384	1.4	18	1,595	1.4	17	560	1.5	19
Oxytetracycline 250mg tablets	1,292	1.3	19	1,416	1.3	20	430	1.1	22
Cefalexin 250mg capsules	1,241	1.2	20	1,661	1.5	16	522	1.4	20
Ciprofloxacin 500mg tablets	1,236	1.2	21	1,724	1.5	15	662	1.8	16
Amoxil 500mg capsules (GlaxoSmithKline UK Ltd)	1,193	1.2	22	587	0.5	33	283	0.8	29
Flucloxacillin 250mg capsules	1,147	1.1	23	1,763	1.6	14	746	2.0	13
Phenoxymethylpenicillin 125mg/5ml oral solution	1,081	1.1	24	712	0.6	27	354	0.9	26
Amoxicillin 250mg capsules	1,073	1.1	25	1,397	1.2	21	575	1.5	18

For peer review only

Table 2: Medical coding of antibiotic prescriptions. Figures are frequencies (percent of unique prescription dates).

	CPRD Aurum	CPRD GOLD	CPRD GOLD England
Number of prescription items	101,360	112,931	37,551
Number of prescriptions with unique date	98,727	108,397	36,617
Medical code recorded on same date	72,989 (74.0)	84,756 (78.2)	28,471 (77.8)
No medical code recorded on same date	25,738 (26.0)	23,641 (21.8)	8,146 (22.2)
Respiratory infection	21,350 (21.6)	26,005 (24.0)	9,549 (26.1)
Genito-urinary infection	11,126 (11.3)	8,762 (8.1)	3,315 (9.1)
Skin infection	6,559 (6.6)	6,559 (6.1)	2,617 (7.1)
Other codes	33,954 (34.4)	43,430 (40.1)	12,990 (35.5)

For peer review only

For peer review only - http://bmjopen.bmj.com/site/about/guidelines.xhtml

Legend for Figure 1: Antibiotic prescribing rates in CPRD Aurum and CPRD GOLD by age-group and sex. Upper figure shows antibiotic prescribing rate per 1,000 patient years; lower figure shows difference in antibiotic prescribing rate per 1,000 patient years.

For peer review only

For peer review only - http://bmjopen.bmj.com/site/about/guidelines.xhtml

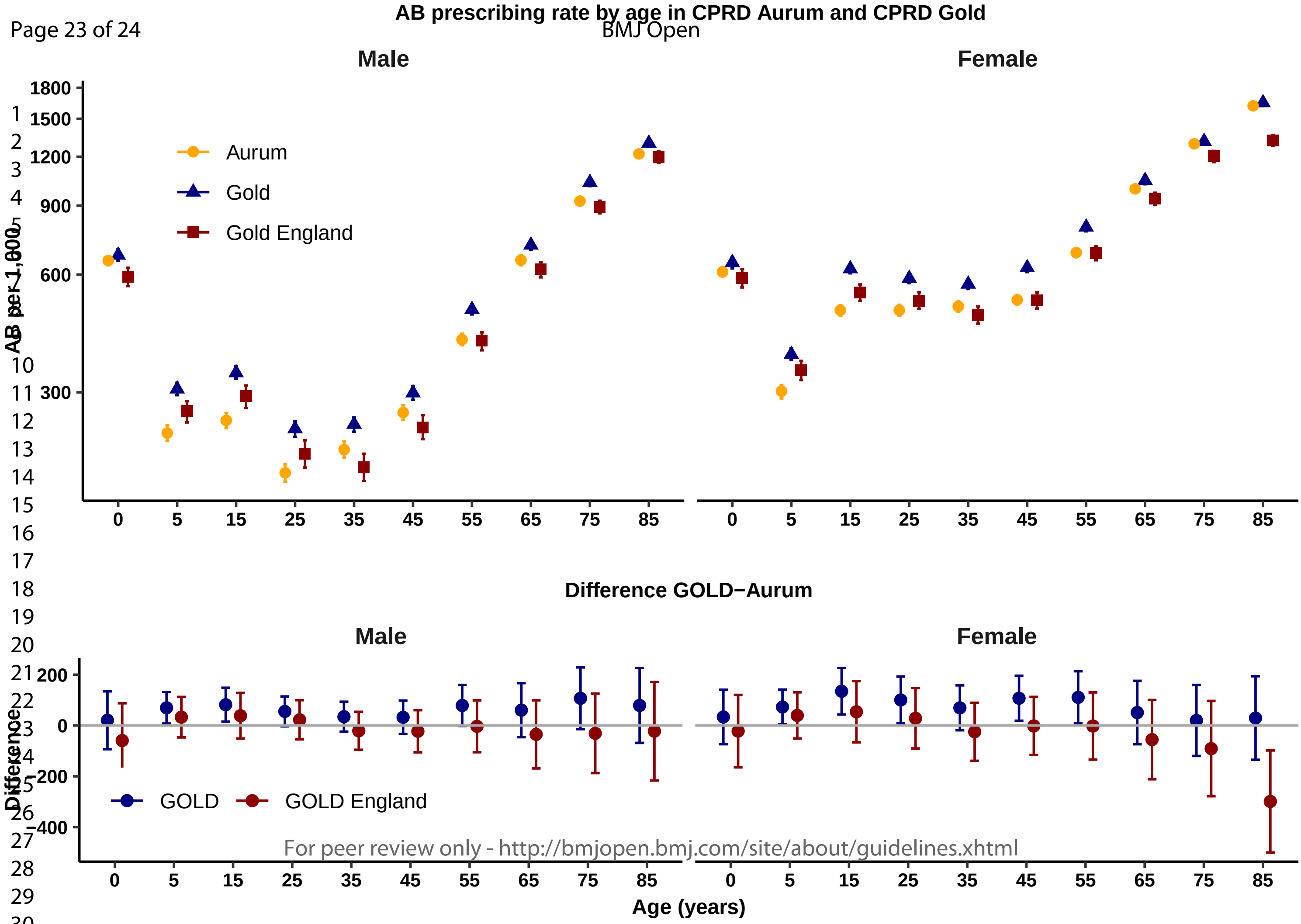
AB prescribing rate by age in CPRD Aurum and CPRD Gold
Male
Female
AB per 1,000
1800
1500
1200
900
600
300
Aurum
Gold
Gold England
0
5
15
25
35
45
55
65
75
85
Difference GOLD−Aurum
Difference
200
0
−200
−400
GOLD
GOLD England
Age (years)

STROBE Statement—Checklist of items that should be included in reports of ***cohort studies***

	Item No	Recommendation
**Title and abstract**	1	(*a*) Indicate the study's design with a commonly used term in the title or the abstract **Abstract line 5**
		(*b*) Provide in the abstract an informative and balanced summary of what was done and what was found **Abstract**
**Introduction**		
Background/rationale	2	Explain the scientific background and rationale for the investigation being reported **P4**
Objectives	3	State specific objectives, including any prespecified hypotheses **P5**
**Methods**		
Study design	4	Present key elements of study design early in the paper **P5-6**
Setting	5	Describe the setting, locations, and relevant dates, including periods of recruitment, exposure, follow-up, and data collection **P5**
Participants	6	(*a*) Give the eligibility criteria, and the sources and methods of selection of participants. Describe methods of follow-up **P5-6**
		(*b*) For matched studies, give matching criteria and number of exposed and unexposed **N/A**
Variables	7	Clearly define all outcomes, exposures, predictors, potential confounders, and effect modifiers. Give diagnostic criteria, if applicable **P6-7**
Data sources/ measurement	8*	For each variable of interest, give sources of data and details of methods of assessment (measurement). Describe comparability of assessment methods if there is more than one group **P6-7**
Bias	9	Describe any efforts to address potential sources of bias **P8**
Study size	10	Explain how the study size was arrived at **P5-6**
Quantitative variables	11	Explain how quantitative variables were handled in the analyses. If applicable, describe which groupings were chosen and why **P6**
Statistical methods	12	(*a*) Describe all statistical methods, including those used to control for confounding **P8**
		(*b*) Describe any methods used to examine subgroups and interactions **N/A**
		(*c*) Explain how missing data were addressed **N/A**
		(*d*) If applicable, explain how loss to follow-up was addressed **N/A**
		(*e*) Describe any sensitivity analyses **N/A**
**Results**		
Participants	13*	(a) Report numbers of individuals at each stage of study—eg numbers potentially eligible, examined for eligibility, confirmed eligible, included in the study, completing follow-up, and analysed **P8**
		(b) Give reasons for non-participation at each stage **N/A**
		(c) Consider use of a flow diagram **N/A**
Descriptive data	14*	(a) Give characteristics of study participants (eg demographic, clinical, social) and information on exposures and potential confounders **P8**
		(b) Indicate number of participants with missing data for each variable of interest **Table 2**
		(c) Summarise follow-up time (eg, average and total amount) **P8**
Outcome data	15*	Report numbers of outcome events or summary measures over time **N/A**
Main results	16	(*a*) Give unadjusted estimates and, if applicable, confounder-adjusted estimates and their precision (eg, 95% confidence interval). Make clear which confounders were

For peer review only - http://bmjopen.bmj.com/site/about/guidelines.xhtml
For peer review only

		adjusted for and why they were included **N/A**
		(*b*) Report category boundaries when continuous variables were categorized **P7**
		(*c*) If relevant, consider translating estimates of relative risk into absolute risk for a meaningful time period **N/A**
Other analyses	17	Report other analyses done—eg analyses of subgroups and interactions, and sensitivity analyses **N/A**
**Discussion**		
Key results	18	Summarise key results with reference to study objectives **P10**
Limitations	19	Discuss limitations of the study, taking into account sources of potential bias or imprecision. Discuss both direction and magnitude of any potential bias **P11**
Interpretation	20	Give a cautious overall interpretation of results considering objectives, limitations, multiplicity of analyses, results from similar studies, and other relevant evidence **P12-13**
Generalisability	21	Discuss the generalisability (external validity) of the study results **P13**
**Other information**		
Funding	22	Give the source of funding and the role of the funders for the present study and, if applicable, for the original study on which the present article is based **P13**

*Give information separately for exposed and unexposed groups.

Note: An Explanation and Elaboration article discusses each checklist item and gives methodological background and published examples of transparent reporting. The STROBE checklist is best used in conjunction with this article (freely available on the Web sites of PLoS Medicine at http://www.plosmedicine.org/, Annals of Internal Medicine at http://www.annals.org/, and Epidemiology at http://www.epidem.com/). Information on the STROBE Initiative is available at http://www.strobe-statement.org.

For peer review only
